# Guideline on *management* of the acute asthma attack in children by Italian Society of Pediatrics

**DOI:** 10.1186/s13052-018-0481-1

**Published:** 2018-04-06

**Authors:** Luciana Indinnimeo, Elena Chiappini, Michele Miraglia del Giudice, The Italian Panel for the management of acute asthma attack in children Roberto Bernardini, Carlo Capristo, Fabio Cardinale, Salvatore Cazzato, Giampiero Chiamenti, Iolanda Chinellato, Giovanni Corsello, Renato Cutrera, Liviana Da Dalt, Marzia Duse, Filippo Festini, Sandra Frateiacci, Domenico Minasi, Andrea Novelli, Giorgio Piacentini, Pietro Scoppi, Eleonora Tappi

**Affiliations:** 1 Pediatric Department “Sapienza” University of Rome, Policlinico Umberto I Viale Regina Elena 324, 00161 Rome, Italy; 2 Pediatric Infectious Disease Unit, Anna Meyer Children’s University Hospital, Florence, Italy; 3 Department of Woman and Child and General and Specialized Surgery, University of Campania Luigi Vanvitelli, Naples, Italy

**Keywords:** Asthma, Asthma attack, Children, Guidelines

## Abstract

**Background:**

Acute asthma attack is a frequent condition in children. It is one of the most common reasons for emergency department (ED) visit and hospitalization. Appropriate care is fundamental, considering both the high prevalence of asthma in children, and its life-threatening risks.

Italian Society of Pediatrics recently issued a guideline on the management of acute asthma attack in children over age 2, in ambulatory and emergency department settings.

**Methods:**

The Grading of Recommendations Assessment, Development, and Evaluation (GRADE) methodology was adopted. A literature search was performed using the Cochrane Library and Medline/PubMed databases, retrieving studies in English or Italian and including children over age 2 year.

**Results:**

Inhaled ß_2_ agonists are the first line drugs for acute asthma attack in children. Ipratropium bromide should be added in moderate/severe attacks. Early use of systemic steroids is associated with reduced risk of ED visits and hospitalization. High doses of inhaled steroids should not replace systemic steroids. Aminophylline use should be avoided in mild/moderate attacks. Weak evidence supports its use in life-threatening attacks. Epinephrine should not be used in the treatment of acute asthma for its lower cost / benefit ratio, compared to β_2_ agonists. Intravenous magnesium solphate could be used in children with severe attacks and/or forced expiratory volume1 (FEV1) lower than 60% predicted, unresponsive to initial inhaled therapy. Heliox could be administered in life-threatening attacks. Leukotriene receptor antagonists are not recommended.

**Conclusions:**

This Guideline is expected to be a useful resource in managing acute asthma attacks in children over age 2.

## Background

Acute asthma attack is a frequent condition in children. It is one of the most common reasons for emergency department (ED) visits and hospitalization [1]. It can be triggered by viral infections, atypical bacteria (i.e. *Mycoplasma pneumoniae*) infections, allergens and/or air pollutants, including tobacco smoke, medications, physical exercise, and stress and emotions [1]. Acute asthma attack can occur as a first episode in undiagnosed children or in children with a previous asthma diagnosis and an uncontrolled disease despite therapy [2]. Indeed, despite advances in therapy, asthma remains a disease that is not optimally controlled in many children [2]. Asthma attacks can be particularly recurrent or life-threatening and increasingly expensive in unresponsive children [2].

The multidisciplinary ISP panel recently issued a new guideline on the management of acute asthma attack in children over age 2, in ambulatory and ED settings, using the GRADE methodology [3]. The guideline aims to deliver up to date scientific evidence and recommendations to pediatricians, general practitioners, Emergency Medicine Physicians, and nurses.

## Methods

This Guideline was issued by the ISP, jointly with the Italian Society of Pediatric Respiratory Diseases, the Italian Society of Pediatric Immunology and Allergology, and the Italian Society of Pediatric Emergency Medicine. The document was developed by a multidisciplinary panel of clinicians and experts in evidence-based medicine who were identified with the help of the participating scientific societies. Specifically, the panel included experts in the fields of general pediatrics, emergency medicine, epidemiology, nursing practice, pharmacology, research methodology, and a member of the parents’ association FEDERASMA. No panel member declared any conflict of interest.

The panel met in two occasions, and many of the consultations involved in the guideline development and draft processes took place interactively by e-mail or phone. The panel members first defined the objectives of the guideline, the essential clinical questions, and the appropriate inclusion and exclusion criteria for the studies from which evidence would be derived. They also identified the information sources and biomedical databases that would be consulted, and the search terms that would be used in constructing the search strategy.

The objective of the guideline was to optimize the management of acute asthma attack in children over age 2, in ambulatory and emergency department settings. This guideline was not intended for children aged 2 years or younger, with acquired or congenital immunodeficiency, major pre-existing, chronic heart or lung disease, and should not be used to treat children admitted to hospital ward or to intensive care unit (ICU).

The quality of evidence and strength of recommendations were rated using the Grading of Recommendation Assessment, Development, and Evaluation (GRADE) approach [3].

### Literature search

Literature search was performed using the Cochrane Library and Medline/PubMed databases, using appropriated key words and retrieving studies published between January 2009 and December 2016, including children aged more than 2 years. The results of this search were then evaluated and selected based on both methodology and relevance. An updated literature search was performed before preparing the final draft; this search identified no additional relevant publications.

### Study selection, levels of evidence, and strength of recommendations

The selection of studies, data extraction and quality assessment were performed by specially trained personnel, following the GRADE methodology [3]. Briefly, evidence was evaluated according to six categories: 1) risk of bias, 2) inconsistency, 3) indirectness, 4) imprecision, 5) publication bias, and 6) other criteria. Quality of the studies can be up- or down-graded due to magnitude factors, limitations in any of the aforementioned categories or other factors [3]. Finally, 4 levels of quality of evidence were indicated (high, moderate, low, very low). Subsequently, balances were assessed between benefit and harm, patients’ values and preferences, cost and resources, and feasibility and acceptability of the intervention, and recommendations were formulated considering 4 grades of strength (Positive-strong; Positive-weak; Negative -strong; Negative-weak) [3]. A strong recommendation was worded as “we recommend” or “it should…” and a weak recommendation as “we suggest” or “it could…” The full text of the guidelines and all the related documents are available at the website of the ISP (www.sip.it).

## Results

### Clinical and objective assessment

History should be collected very carefully since it is an extremely important tool to predict the severity of exacerbations and the risk for hospitalization. Symptoms are poorly related to the severity of airway obstruction. Therefore, objective evaluations (i.e. pulse oximetry; peak expiratory flow; FEV1; blood gas measurement) should be considered [4–14]. However, the value of pulmonary function parameters in the assessment of patients with respiratory distress is modest [4–15]. Only three high quality studies are available [11–13]. One is a systematic review of 60 studies showing that none of the available score are validated in the clinical practice [11]. The other one is an observational prospective study including 101 children, aged > 6 years, demonstrating that the Clinical Asthma Score was not related to the spirometry results [12]. More recently Eggink and collaborators performed a prospective, high quality study, reviewed and validated clinical scores for dyspnoea severity in children, and authors concluded that the commonly used dyspnoea scores have insufficient validity and reliability to allow for clinical use without caution [13].

Levels of severity of the acute asthma attack and indications for hospitalization are summarized in Tables 1 and 2. It should be underlined considering that low oxygen saturation, especially after initial bronchodilator treatment, allows the identification of patients with more severe asthma [2, 16, 17]. Respiratory physiology studies showed that in mild acute asthma attack, PaCO2 values are usually normal. Increasing values of PaCO2 may be an ominous sign of impending respiratory failure, in presence of respiratory distress [2, 16, 17].

**Table 1:**
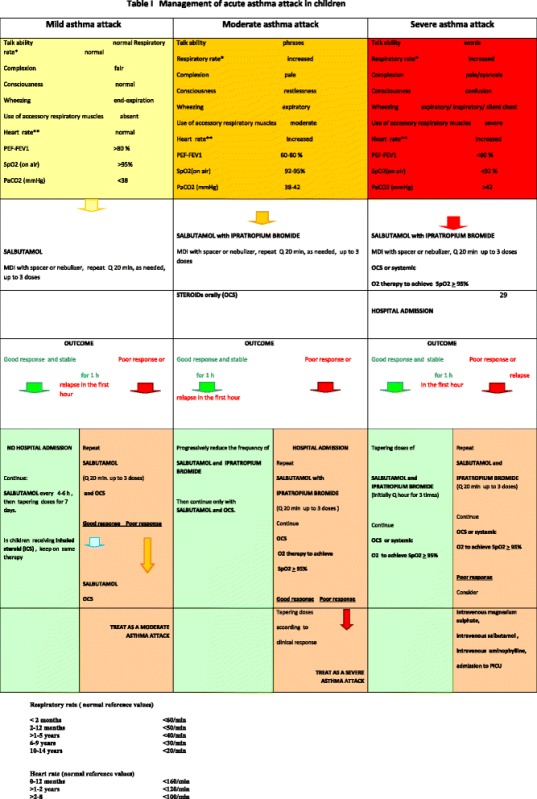
Management of acute asthma attack in children

Note. PEF is expressed as percentage of personal best. Not all parameters have to be abnormal, but a single abnormality may be sufficient to classify a patient into a severity class. The severity category may change when more information is available or over time

**Table 2:** Conditions requiring hospitalization

Signs and symptoms of acute respiratory failure Worsening of clinical parameters after bronchodilator treatment SatO2 < 92% after bronchodilator treatment PEF < 60% predicted after bronchodilator treatment	
Concomitant complications (pneumothorax, atelectasies, pneumonia)	
Severe asthma itself, irrespective of worsening; History of previous severe life-threatening asthma episodes, or previous admission to ICU	

### Recommendation

Level of severity should be assessed considering both clinical and objective evaluations, including pulse oximetry, peak expiratory flow or FEV1. Blood gas measurement should be reserved only to more severe attacks.

### Positive strong recommendation

#### Treatments

##### Oxygen

Numerous studies have confirmed that hypoxia is almost always present during acute asthma attack, its degree depending on the severity of the episode [2, 14–17]. Therefore monitoring the blood oxygenation level, mainly through pulse oxymetry, is fundamental in order to select children who deserve oxygen therapy. Oxygen saturation should be obtained when the patient is breathing room air. However, it is not necessary to cease oxygen therapy to measure pulse oximetry, if it has already been started. Clinical judgment should be applied in any circumstance [2, 16, 17].

### Recommendation

Humidified oxygen therapy using a tight fitting face mask or nasal cannula should be administered to children with severe acute asthma attack and/or SpO_2_ < 92%. Flow rates and oxygen concentration may be released by specific Venturi mask and should be sufficient to achieve saturations of ≥ 95%.

### Positive strong recommendation

#### Inhaled short-acting ß_2_ agonists

Inhaled short-acting ß2 agonists are the first line treatment for acute asthma attack in children. Salbutamol is a useful medication that can be used in children of all ages. Inhaled via is the traditional route of administration [18]. **S**albutamol given continuously via nebulizer was not associated with a better outcome with respect to frequent intermittent administration, in a systematic review, dating back to 2003 and including only one pediatric study [19, 20]. In 2013 Cochrane including 1897 children and 729 adults in 39 trials, Metered-Dose Inhalers (MDI) with spacer was considered the preferred option for delivering ß2 agonists in children with mild to moderate asthma attack [21].

Salbutamol dose to be administered through MDI with spacer should be individualized according to the asthma attack severity: 200–400 μg/dose (2–4 puffs/dose) could be sufficient in mild attacks. Children with severe asthma should receive frequent doses of nebulised bronchodilators (2.5 to 5 mg of salbutamol), driven by oxygen, given the risk of oxygen desaturation while using air-driven compressors. Once improving on two- to four-hourly salbutamol, patients should be switched to a MDI with spacer [16, 17, 22].

### Recommendation

Salbutamol is the first line treatment for acute asthma attack in children. In severe attack it should be administered frequently, up to 3 times every 20–30 min within the first hour.

### Positive strong recommendation

### Recommendation

MDI with spacer should be used to delivery ß2 agonists in children with mild to moderate asthma attack. Children with severe asthma should receive frequent doses of nebulised bronchodilators (2.5 to 5 mg of salbutamol), driven by oxygen.

### Positive strong recommendation

#### Intravenous short-acting ß_2_ agonists

Literature data regarding iv short-acting ß_2_ agonists use are poor. No consistent evidence favoring the use of iv short-acting ß_2_-agonists for patients with acute asthma were evidenced in a 2012 Cochrane including 2 pediatric studies on children (one in ICU) [23].

Some authors suggest the use of iv salbutamol in addition of long-acting ß_2_ agonists in children with severe asthma attack unresponsive to initial therapy [23]. The recommended dose is a single bolus of 15 μg/kg (diluition: 200 μg/mL for central iv line; 10–20 μg/mL for peripheral iv line) over 10 min, followed by continuous infusion of 0.2 μg/kg /min. Higher doses (1–2 μg/kg/min up to 5 μg/kg/min) can be administered in unresponsive children [2, 16]. Intravenous salbutamol should be given in the ICU with continuous ECG and twice daily electrolyte and lactate monitoring [17].

### Recommendation

Salbutamol could be administered intravenously (iv) in children with asthma attack not responding to initial therapy.

### Positive weak recommendation

### Recommendation

Children receiving iv salbutamol should be admitted to intensive care unit with continuous ECG and twice daily electrolyte and lactate monitoring.

### Positive strong recommendation

#### Ipratopium bromide

Ipratopium bromide induces a slower broncodilator response than ß_2_ agonists, but the combination of the two medications produces a synergic effect. In severe attack the recommended nebulized dose is 125–250 μg/dose (in children < 4 years of age) to 250–500 μg/dose (in children ≥ 4 years of age), in combination with nebulized salbutamol. It should be administered frequently, up to 3 times every 20–30 min, within the first hour. The ipratropium dose should be tapered to 4 to 6 hourly or discontinued [17]. Once ipratropium bromide is discontinued, salbutamol dose should be tapered to one- to two-hourly thereafter according to clinical response.

A 2012 Cochrane review [24] including four trials on 173 children found that treatment failure on anticholinergics alone was more likely than when anticholinergics were combined with short-acting ß_2_ agonists (OR 2.65; 95% CI 1.2 to 5.88). Authors concluded that inhaled anticholinergic drugs alone are not appropriate for use as a single agent in children with acute asthma exacerbations. In a subsequent 2013 Cochrane review [25], including 15 studies with 2497 children, the addition of an anticholinergic to a SABA significantly reduced the risk of hospitalization (RR: 0.73; 95% CI: 0.63 to 0.85). Fewer children treated with anticholinergics plus short-acting ß_2_ agonists reported nausea and tremors compared to short-acting ß2 agonists alone; no significant group difference was observed for vomiting. Authors conclude that inhaled anticholinergics given in addition to β2-agonists are effective in reducing hospitalizations in children arriving in ED with a moderate to severe asthma exacerbation [25]. Only one study yielded a different result, however it should be noticed that MDI plus spacer was used [26]. It was a prospective, single-blinded, randomized, controlled, equivalence trial in a tertiary pediatric ED, including 347 children, and showing that the addition of ipratropium bromide was not significantly associated with a reduction in admission rates [26]. In a 2014 Cochrane review, including 4 studies on 472 children admitted to pediatric wards, no evidence of benefit for length of hospital stay nor other markers of response to therapy was noted when nebulised anticholinergics were added to short-acting β2-agonists [27].

### Recommendation

Nebulized inhaled ipratropium bromide, given in addition to short-acting β2-agonists, should be administered in children with a moderate to severe asthma attack.

### Positive strong recommendation

#### Steroids

Systemic steroids (SS) have been reported to be effective in the treatment of acute asthma attack in children, with no difference between oral or intravenously/intramuscle route of administration [28]. Therefore the oral steroids are preferable, in the absence of vomiting. Dexamethasone, prednisone, and prednisolone are equally effective even if dexamethasone is associated with a higher risk of vomiting [28]. A recent open randomized trial [29] and one meta-analysis including 6 pediatric studies [30] demonstrated no different efficacy between prednisone and dexamethasone in children with acute asthma attack. However, this meta-analysis concludes that “emergency physicians should consider single or 2-dose dexamethasone regimens over 5-day prednisone/prednisolone regimens for the treatment of acute asthma exacerbations”, due to easier administration and less side effects with dexamethasone [30]. A recent meta-analysis including 18 studies with a total of 2438 participants assessed the efficacy and safety of any dose or duration of oral steroids versus any other dose or duration of oral steroids for adults and children with an asthma exacerbation [31]. Literature data was not sufficient to discriminate whether shorter or lower-dose regimens are less effective than longer or higher-dose regimens, or indeed more adverse events are associated with the latter. Thus, authors underline that some regimen characteristics including palatability, regimen duration, and costs should be considered in order to improve adherence in individual patients [31]. Another recent meta-analysis, including 10 RCT in children, concluded that dexamethasone is likely to have less adverse effects than others corticosteroids, and similar efficacy in reducing hospitalizations and revisits [32].

Considering the time needed to induce gene expression and protein synthesis, the majority of pharmacological effects of steroid are not immediate, but are evident some hours after their intake. However, glucocorticoids can have rapid effects on inflammation which are not mediated by changes in gene expression [33]. Therefore their efficacy is optimized by an early use. Accordingly, an inverse association between time of administration and risk of hospitalization has been reported in a systematic review [34]. Steroid intake within the first hour from admission to the ED was associated with a significantly reduced time spent in the ED and a lower hospitalization rate [33].

The optimal duration of steroid therapy is unclear, some experts would suggest prolonging this therapy for 3 to 5 days, with no need to taper the dose at the end, particularly using molecules with short or intermediate half- life [34]. In a recent review acute single or recurrent systemic short-term (< 2 weeks) steroids in children with asthma exacerbations did not show any concern about short-term adverse effects [35].

However, it is important to underline the long-term risks caused by recurrent administration of oral steroids in children with asthma. Literature data report that children who require more than four courses of oral corticosteroids as treatment for underlying disease, including asthma, are at increased risk of fracture [36]. Furthermore, the CAMP study demonstrated that multiple oral corticosteroid bursts over a period of years can produce a dose-dependent reduction in bone mineral accretion and increased risk of osteopenia in children with asthma [37].

### Recommendations

Systemic steroids (SS) should be used in the moderate to severe acute asthma attack in order to reduce the hospitalization rate and the risk of recurrence. Oral course, should be preferred in children able to retain drugs orally.

### Positive strong recommendation

#### Inhaled steroids

Six randomized controlled trials, overall including 1302 children, and 4 systematic reviews [38–47] were evidenced through the literature search. Moreover other important studies, although published before 2009, have been considered [48]. Higher clinical efficacy of inhaled high-dose corticosteroids (ICS) with respect to the placebo was observed in one randomized controlled trials (RCT) [38]. The addition of nebulized high-dose budesonide was evaluated in adjunction to standard therapy without oral steroids in children with moderate-to-severe acute asthma exacerbation [38]. Complete remission rate was significantly higher (84.7% vs. 46.3%; *P* = 0.004) and need for oral corticosteroids was significantly lower (16.9% vs. 46.3%, *P* = 0.011) in the group receiving budesonide than in control group [38].

Two RCTs, whose results have been reported in three manuscripts [39–41], showed that addition of high dose ICS to standard asthma attack therapy, including SS, was not associated with clinical improvement after one and 2 h. However, in one study, it was associated with a decreased admission rate of children with severe acute asthma [40].

Two randomized clinical trials compared the effectiveness of high-dose of ICS vs, SS [42, 43], the results showed that ICS and SS have the same efficacy to improve clinical symptoms. However, one study [38] showed that in the group treated with high doses of budesonide (800 μg/ 20 min) there was an increase in the percentage of children discharged from hospital after 2 h compared to the group treated with prednisolone (2 mg/kg).

A systematic review including eight studies published between 1995 and 2006 [44] showed no differences in the treatment with high-dose ICS or SS regarding admission rates, ED visits and rescue medications.

Two Cochrane reviews [45, 46], including both adult and pediatric studies, conclude that there is insufficient evidence that ICS treatment results in clinically important changes in pulmonary function or clinical scores when used in acute asthma in addition to SS [45, 46]. Therefore there is insufficient evidence that ICS therapy can be used in place of SS therapy when treating acute asthma [45, 46]. A 2012 Cochrane Review [47] evaluated the effectiveness of the ICS treatment after discharge from ED and concluded that ICS provides no additional benefit to standard therapy with SS in the post-discharge treatment of children with acute asthma. In conclusion, there was some evidence that high doses of ICS can be as effective as SS in the post-discharge treatment of children with acute asthma. However, it should be noticed that the settings where the trials have been performed - including specifically dedicated nurses and/or doctors - are difficult to replicate in the everyday practice in ED or ambulatory. In such situations, prudently, SS should be preferred. In addition, higher cost of ICS should be considered.

### Recommendation

- High doses of ICS should not be used instead of SS in asthma attack.

### Negative strong recommendation

- Children treated with ICS can continue to use the usual doses of ICS during the asthma attack.

### Positive strong recommendation

#### Aminophylline

Several studies are available comparing the efficacy of aminophylline in different clinical settings (i.e. aminophylline compared to placebo when added to inhaled β2-agonists, or compared to iv salbutamol in more severe attacks) [49]. In a recent review results from 12 RCTs, involving 586 children, and comparing aminophylline with placebo or usual treatment were summarized [49]. Improvement in clinical severity scores was found in 3 RCTs but not confirmed in other six, while 2 RCTs showed improved lung function scores and two did not [49]. One trial showed that iv aminophylline reduced ICU admission rates, but no trial evidenced any benefit of aminophylline on length of hospital or ICU stay [49]. Seven out of these 12 trials have been included in a 2005 Cochrane review [50]. This review concluded that intravenous aminophylline improved lung function within 6 h of treatment, but did not appear to reduce symptoms or length of hospital stay, and there was insufficient evidence to evaluate its impact on ICU rates [50]. In conclusion, in the setting of moderate asthma attack, the association of aminophylline to inhaled β2 agonists and steroids in acute asthma does not offer substantial benefits [49, 50].

In the setting of severe asthma attacks data of the literature comparing iv salbutamol with iv aminophylline are poor [49, 51], and no substantial difference of efficacy emerges between the two drugs. In particular, iv aminophylline and salbutamol (or terbutaline) have been compared, head-to-head, in 4 RCTs including 202 children [49]. In three trials no different clinical severity scores were reported between iv salbutamol and iv aminophylline. Moreover no difference was observed in the one study reporting ICU admission rates and in two RCTs reporting length of hospital stay [49]. No study reported lung function outcomes. These paediatric studies have been included in a subgroup analysis in a Cochrane review [51], concluding that there was no consistent evidence to help decide between iv aminophylline and iv salbutamol as therapy of choice. In a recent study, a single i.v. dose of magnesium sulphate, added to inhaled β2 agonist and SS, was more useful and safe than iv aminophylline in 100 children with severe acute asthma [52]. In summary, the administration of iv aminophylline can be considered in addition to usual care in patients with impending respiratory failure and in those who have shown a good response to the drug in the past [2, 16, 17]. Serum levels measurements are needed, especially in patients already being treated with oral aminophylline [2, 16, 17]. Few studies are available regarding the use of low dose of aminophylline but further data are needed regarding this issue [53].

### Recommendation

Aminophylline should not be used in mild to moderate acute asthma.

### Negative strong recommendation

### Recommendation

Iv Salbutamol or iv aminophylline could be used in severe acute asthma in children non-responder to inhaled β_2_ agonist and oral corticosteroids. There are no significant differences between the two treatments.

### Positive weak recommendation

#### Epinephrine

Epinephrine does not offer any advantages compared to β_2_ agonist in the treatment of acute asthma and is associated with a greater risk of side effects, especially in hypoxemic patients. Epinephrine could be used if β_2_agonists are not available [2, 16, 17].

### Recommendation

Epinephrine should not be used in the treatment of acute asthma for its lower cost / benefit ratio, compared to β_2_ agonists.

### Negative strong recommendation

#### Magnesium sulphate

The childhood experiences are still limited and related to the use of a single dose of 25–40 mg/kg iv. In a recent RCT of moderate quality [54] in 143 children with severe asthma, the intravenous administration of magnesium sulphate during the first hour was associated to a significant decrease in the number of patients who required mechanical ventilation. In a pharmacokinetic study [55] in 19 children with severe asthma, a bolus of magnesium sulphate (50–75 mg/kg), followed by continuous infusion (40 mg/kg/h) for 4 h, was safe and maintained appropriated levels of Mg in serum.

There are conflicting data about the use of nebulized MgSO4 in addition to β_2_ agonists in asthma exacerbations [56, 57].

One RCT that included 508 children with severe acute asthma [58] compared the effect of nebulized magnesium sulphate to placebo. In the treated group there was a statistically significant improvement in asthma score after 60 and 240 min. However, the clinical relevance of this finding is uncertain. No serious adverse event was observed in 19% of patients in the Mg group and in 20% of the controls. Moreover, the study concludes there might be a role for nebulised MgSO4 in children with a severe exacerbation whose SaO2 in air after the first nebulised treatment remains below 92%, and in those with a shorter duration of symptoms [58]. Similarly a role of nebulised MgSO4 has been considered by other authors [59], but further studies are needed at this regard.

A recent RCT evaluated the effect of nebulized MgSO4, on FEV1 and PEF in children with asthma induced by acetylcholine [60]. The nebulized MgSO4 showed a wide bronchodilator effect but the rise in FEV1 and PEF was not superior to salbutamol. There is no evidence to support that the combination of salbutamol and magnesium sulphate displays a synergistic effect. No significant adverse event risk was reported.

A recent meta-analysis [61] including 5 studies (182 children) demonstrated that treatment with iv MgSO4 reduced the odds of admission to hospital by 68%. Adverse events have not been reported consistently with magnesium sulphate therapy.

### Recommendation

MgSO4 could be used intravenously in children with severe asthma not responding to the initial treatment. MgSO4 could be also used if FEV1 is less than 60% predicted, after the first hour.

### Positive weak recommendation

### Recommendation

Nebulized MgSO4 should not be used in mild, moderate or severe asthma, since the available evidence is poor.

### Negative strong recommendation

#### Heliox

A gas mixture containing helium / oxygen (Heliox) can decrease respiratory failure and improve ventilation in patients with airway obstruction. The use of this mixture is not indicated in mild-moderate asthma. It can be used as an alternative to oxygen in severe asthma not responding to the initial treatment [62].

According to results of a systematic review of 5 pediatric RCT (1996–2010) and 143 children, there are insufficient data to support the routine use of heliox in acute asthma. In particular, not benefits in terms of rate/length of hospitalization, nor percentage of children requiring intubation have been demonstrated [63]. However, it is a safe therapy, and some data suggest that it may be beneficial to patients with severely impaired lung function. A systematic review and meta-analysis [64], including 3 pediatric studies and 113 children, showed that heliox used as a vehicle to deliver β_2_ agonist (compared to oxygen) was associated with improvement of acute asthma, especially in most severe attacks. It also was associated with reduced need for hospitalization [64].

Notably, to administer the heliox, a non-rebreathing high-flow system is needed. Heliox needs a high flow of oxygen to the appropriate sized particles.

### Recommendation

A helium-oxygen mixture (70%: 30%) could be used in severe asthma unresponsive to standard therapy.

### Positive weak recommendation

#### Leukotriene modifiers

A Cochrane review was available including 1470 adults and 470 children (aged 2–12), treated for acute asthma in ED and randomized to receive montelukast or placebo in addition to standard therapy [65]. No statistically significant difference was found in the risk of hospitalization with the use of oral montelukast in addition to standard therapy [65]. These results have been recently confirmed by Wang and colleagues in one trial comparing montelukast versus placebo in 117 children, aged 2 to 5 years, demonstrating no difference in PEF and lung function improvement [66].

### Recommendation

Leukotriene modifiers in addition to standard therapy should not be used.

### Negative strong recommendation

## Conclusions

This guideline is an updated tool for the management of acute asthma attack in children over age 2. The review of the literature supports the use of salbutamol as the most appropriate β_2_ agonist. Adding ipratropium bromide is an effective aid in moderate and severe attack. Oral corticosteroids should be used in moderate-to-severe acute asthma attacks to prevent hospitalizations and symptom relapse. Adding steroids to the moderate and severe attacks is more effective if administered at an early stage. Intravenous steroids should be reserved for selected children who are unable to take oral medications. High doses of inhaled steroids should not replace systemic steroids. Aminophylline use is not recommended in mild to moderate acute asthma attacks. Weak evidence supports its use in life-threatening attacks.Epinephrine should not be used in the treatment of acute asthma for its lower cost / benefit ratio, compared to β_2_ agonists. The use of iv MgSO4 could be considered only in children with severe asthma attack who are unresponsive to initial treatment and/or who have FEV1 less than 60% predicted, after 1 h of standard therapy. Helium-oxygen mixture (70%:30%) can be used in severe asthma attack unresponsive to standard therapy. Leukotriene modifiers are not currently recommended.
